# Cancer screening learning for adults with intellectual disability

**DOI:** 10.17269/s41997-025-01105-6

**Published:** 2026-04-29

**Authors:** Daniel Satgé, Elodie Neumann-Michel, Amaëlle Otandault, Anaïs Lecluse, Elisangela Olivier, Marc Palpacuer, Xavier Heber-Suffrin, Brigitte Trétarre, Chris Serrand, Geneviève Petitpierre

**Affiliations:** 1 ONCODEFI Parc Euromédecine, Montpellier, France; 2 Desbrest Institute of Epidemiology and Public Health, UMR UA11, INSERM, University of Montpellier, Montpellier, France; 3 Établissement et Service d’Aide par le Travail L’Envol, UNAPEI 34, Castelnau-Le-Lez, France; 4 Association « Nous Aussi », Vétraz-Monthoux, France; 5 Herault Tumor Registry, Montpellier, France; 6 UMR 1295, CERPOP Constitutive team, Inserm, University of Toulouse III Paul Sabatier, Toulouse, France; 7 Département de Biostatistique, Épidémiologie Clinique, Santé Publique Et Innovation Méthodologique, CHU Nîmes, Nîmes, France; 8 Department of Special Education, University of Fribourg, Fribourg, Switzerland

**Keywords:** Accessible information, Cancer screening, Intellectual disability, Learning, Interventional study, Information accessible, Dépistage du cancer, Déficience intellectuelle, Apprentissage, Étude interventionnelle

## Abstract

**Objectives:**

Compared to the general population, persons with intellectual disability (ID) have a similar cancer rate, but cancer is often discovered at a later stage. Adults with ID show lower participation in organized screenings for breast, cervical, and colorectal cancer. Here we tested an intervention to increase cancer screening knowledge and intention to participate among persons with ID.

**Methods:**

An open-label cluster randomized controlled trial was co-constructed with people having ID. Participants with ID underwent interventions about cancer screening or oral hygiene, using PowerPoint presentations, booklets, dice games, workshops, films, and discussions. Both groups completed a questionnaire two weeks before the intervention and at 15 min, three months, and one year after the intervention, which evaluated their knowledge gain and intention to participate in cancer screenings.

**Results:**

At 15 min, three months, and one year after the intervention, participants in the cancer group showed significantly improved cancer screening knowledge (*p* < 0.001). The intention to participate in screenings was increased on the intervention date (*p* < 0.001), but this change was non-significant three months later, and observed as a trend at one year (*p* = 0.068). A steering group of persons with ID gave advice regarding participant recruitment, conducting sessions, and modifying the film scenario, PowerPoint presentation, and questionnaire. Persons with ID co-constructed the booklet on cancer screening and acted in the film.

**Conclusion:**

Participation of persons with ID greatly improved the study efficacy. This research provides strong evidence supporting direct interventions for people with intellectual disability to increase their participation in organized cancer screening.

**Supplementary Information:**

The online version contains supplementary material available at 10.17269/s41997-025-01105-6.

## Résumé

**Objectifs:**

Les personnes qui ont une déficience intellectuelle (DI) développent autant de cancers que les personnes dans la population générale. Ces cancers sont souvent découverts à un stade avancé. Les adultes atteints de DI participent peu au dépistage organisé des cancers du sein, du col de l’utérus et colorectal. Nous avons testé une intervention visant à accroître les connaissances sur le dépistage des cancers et l’intention d’y participer chez les personnes qui ont une DI.

**Méthodes:**

Un essai contrôlé randomisé en grappes en ouvert a été co-construit avec des personnes DI. Les participants atteints de DI ont bénéficié d’une intervention sur le dépistage, ou bien sur l’hygiène bucco-dentaire. Elle comportait une présentation par diaporama, un livret, un jeu de dés, des ateliers, un film et des discussions. Un questionnaire évaluant d’une part les connaissances, d’autre part les intentions de participer au dépistage des cancers a été complété deux semaines avant l’intervention, puis 15 min, trois mois et un an après l’intervention.

**Résultats:**

Quinze minutes, trois mois et un an après l’intervention, les participants du groupe cancer avaient significativement amélioré leurs connaissances sur le dépistage des cancers (*p* < 0,001). L’intention de participer au dépistage a augmenté le jour de l’intervention (*p* < 0,001), cette modification n’était plus significative trois mois plus tard, mais subsistait cependant à un an comme une tendance (*p* = 0,068). Un comité de pilotage incluant des personnes DI a donné des conseils pour le recrutement des participants, la conduite des séances, pour modifier le scénario du film, les présentations par diaporamas et le questionnaire. Des personnes avec DI ont co-construit le livret de dépistage du cancer et ont été acteurs dans le film.

**Conclusion:**

La participation de personnes qui ont une DI a grandement amélioré l’efficacité de l’étude. Cette étude fournit des arguments solides pour développer des interventions directes auprès de ces personnes afin d’accroître leur participation au dépistage organisé des cancers.

## Introduction

Individuals with intellectual disability (ID) constitute 1 − 2% of the general population and develop cancer as frequently as persons in the general population (Sullivan et al., 2004; Liu et al., 2021). However, in persons with ID, cancers are often discovered at a later stage (Satgé et al., 2014; Heslop et al., 2022; Mahar et al., 2024), complicating treatment and reducing the likelihood of recovery. A study evaluated cancer distribution and diagnosis delays among persons with ID and reported that 12 of 14 adults with colorectal cancer were diagnosed very late, and 11 died of the disease (median survival, 2 months) (Satgé et al., 2023).

Among persons without ID, cancers are mainly discovered due to pain and somatic signs. However, in persons with ID, health professionals often misattribute symptoms to mental disorders (Tuffrey-Wijne, 2010). Cancer screening is an important opportunity for early tumor detection, to enable less burdensome and more successful treatment. Notably, the cancers targeted by national organized screenings—breast and colorectal carcinomas—are those that occur most frequently among adults with ID (Tretarre et al., 2017; Sullivan et al., 2004). Additionally, increased frequencies of sexual relations and unsafe sex in young adults with mild/moderate ID have led to more common high-grade preneoplastic lesions of the uterine cervix (Baines et al., 2018).

Despite these issues, adults with ID are less likely to undergo cancer screening, compared to non-disabled persons (Swaine et al., 2013; Ouellette-Kuntz et al., 2015; Willis et al., 2008)*.* Assistance from professional carers only partly corrects this participation deficit, possibly due to the carers’ fear of and limited knowledge about cancer (Hanna et al., 2011; Wyatt & Talbot, 2013). Persons with ID show interest in their health and exhibit improved psychological well-being when engaged in their own care (Flynn et al., 2016). Since, by definition, people with ID have cognitive limitations, it is crucial to evaluate the extent to which they can understand the cancer screening process.

Few studies have reported interventions designed to directly enhance cancer screening participation among adults with ID. Among three relevant randomized control trials (RCTs) (Elmadani et al., 2024) two have focused on breast and cervical cancer (Parish et al., 2012; Swaine et al., 2014) and one on testicular cancer (Wilson et al., 2018). One evaluation study has focused on cancer screening programs (Howieson & Clarke, 2013), and another on cancer awareness (Gilbert et al., 2007). One study investigated mammograph feasibility (Greenwood et al., 2014). However, these studies have not examined whether persons with ID understand and remember information related to cancer, particularly regarding cancer screening opportunities.

With the aim of protecting them, people with ID have been excluded from research. This has limited the prospects of improving quality of life for this population and reduced their opportunities to contribute to society via research participation (McDonald et al., 2016). Non-biomedical research demonstrates the feasibility and impact of respectfully engaging people with ID in scientific processes (McDonald et al., 2016). Moreover, failure to engage persons with ID in research can limit the meaningful use of scientific advances among this community (Shogren, 2023). Research involving people with ID can reflect their insider cultural knowledge and elucidate how to best improve participant comfort (Kim et al., 2022).

In the present study, we performed a two-arm cluster RCT, with the aim of evaluating whether adults with mild and moderate ID could benefit from a health learning intervention about cancer screening and decide to participate in organized cancer screening opportunities. This study was conducted in an inclusive manner via codesign.

## Methods

### Study design

We performed an open-label, two-arm, cluster randomized controlled trial. Participants with ID received a health education intervention about either cancer screening or dental hygiene, and these two groups were compared. The effects on cancer knowledge were measured 15 days before intervention, the day of intervention, at 3 months, and 1 year post-intervention. This research was authorized by the Commission Nationale Informatique et Libertés (CNIL) Recherche en santé (XTz2999949Y), by the Comité de protection des personnes (CPP) (N° ID-RCB 2022 A00225-38), and by the directors of the medico-social establishments involved in the study.

### Inclusion criteria and recruitment

We recruited 608 participants from 38 medico-social institutions in the departments of Hérault, Gard, and Aude in the South of France, in 2022. Eligibility criteria were age over 20 years, interest in participating, mild or moderate ID, speaking French, and residing or working in a medico-social institution. ID level was indicated in the patient’s chart at the institution. Persons with severe ID and persons with cognitive impairment discovered after 18 years of age were excluded.

Several calls for study participation were organized in the 38 institutions, during which those interested in participating were invited to ask questions about the project and sign a consent document. These sessions were organized with the help of professional caregivers or nurses working in these institutions.

**Interventions** (see also Petitpierre et al., 2025).

All participants were randomized (1:1) to receive one of the two interventions (Supplementary methods). The experimental group received information about cancer screening, while the control group received information about dental hygiene. The main common didactic principles of these interventions have been extensively described in a previous article (Petitpierre et al., 2025) (Supplementary methods).

### The cancer screening instruction module

**Fig. 1:**
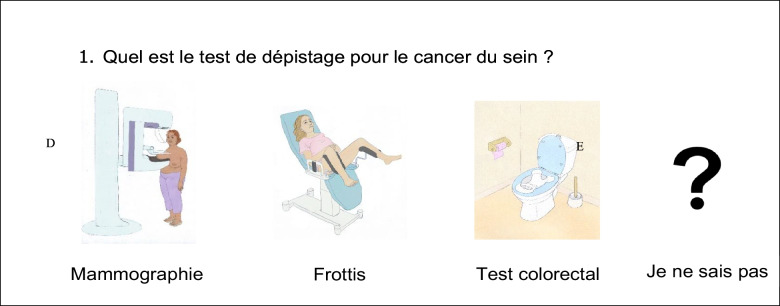
Part of the questionnaire built with images from the screening booklet and PowerPoint. The question is: What is the screening test for breast cancer?

The cancer screening instruction module was co-constructed with people with ID (Petitpierre et al., 2025). It comprised five components using easy-toto-read and understand language: 1) a 20-minute slideshow explaining cancer and organized screenings for breast, colon-rectum, and uterine cervix cancer; 2) a workshop including the colorectal screening kit, a recap dice game with cards (created by EN), and an anatomical breast module for women; 3) a booklet describing scenarios of cancer screening through 47 short texts and images; 4) a five-minute film in which people with ID explain cancer screening to each other, and advise participation in cancer screening; and 5) a 20-item questionnaire to evaluate participants’ knowledge at each session and a recall of the message. Notably, the same pictures were used in the slideshow, the dice game, the booklet, and the questionnaire (Fig. 1).

**The intervention focusing on dental hygiene** (see also supplement 1).

The dental hygiene intervention was largely parallel to the cancer screening intervention. It also included a short theoretical presentation (about the importance of tooth brushing, instead of cancer screening), followed by a workshop and discussion in sub-groups. Control group participants were given a small booklet about dental hygiene to take home after the intervention. They were also shown a short film reminding them of the dental hygiene intervention on D0. The cancer screening and dental hygiene interventions were roughly the same length (1.5–2 h).

### Sessions program

**Fig. 2:**
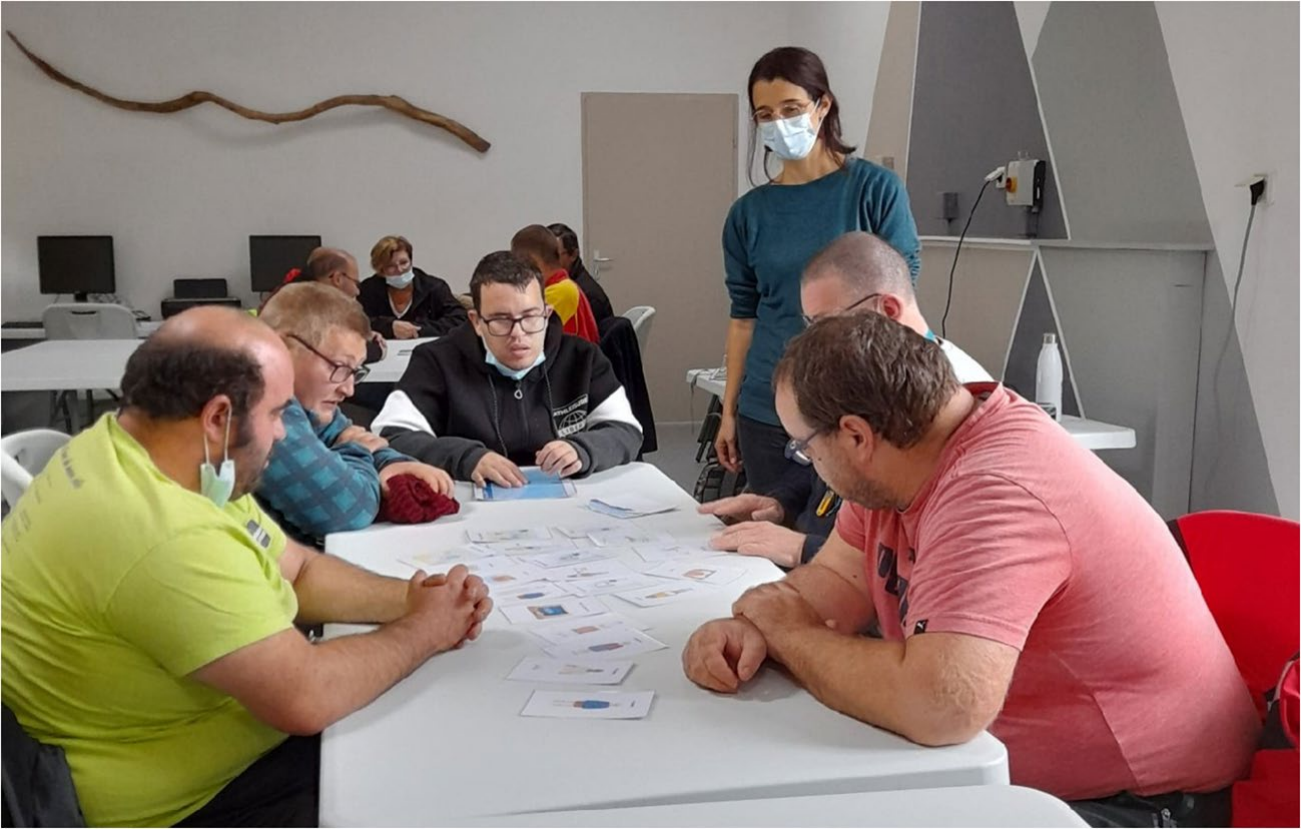
During the workshop, participants play an educational dice game that uses the same images as those in the prevention booklet and in the PowerPoint. One co-author (EN) who created the game is standing near the table

Participants in both groups attended four sessions: 15 days before the training (D-15 days), on the training day (D0), three months after the training (D + 3 months), and one year after the training (D + 1 year). All sessions took place at the institutions, and each lasted between 1.5 to 2 h. They were led by two members of the research team, in the presence of at least one caregiver from the institution (Fig. 2). At every stage, the moderators paid particular attention to the participants’ expressions and emotions. This was essential because discussing cancer screening can trigger memories for participants who have been affected by cancer, through family members or acquaintances.

**Fig. 3:**
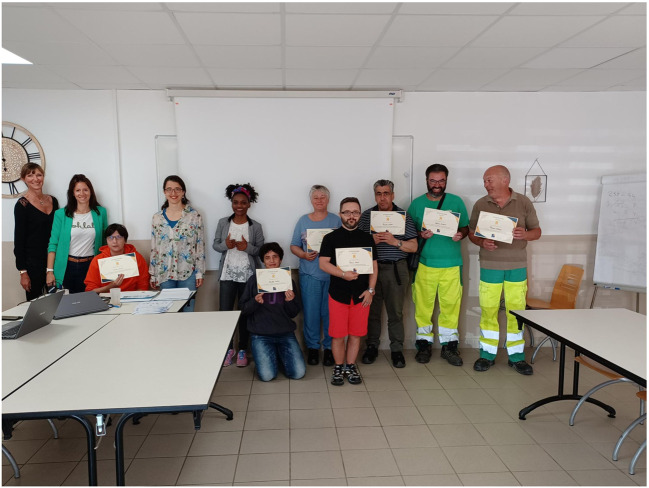
Participants showing their diplomas during the last session at 1 year

At the first session (D-15 days), the study was presented and explained to a group of 8–12 participants. Next, the participants completed the questionnaire to establish baseline knowledge and intentions regarding screening. At the second session (D0), for each study arm, a slideshow was presented, followed by a workshop and discussion. At the end of this session, participants completed the questionnaire for the second time. The third session (D + 3 months) began with the questionnaire. This was followed by the 5-min film (on cancer and screening or oral hygiene), which introduced a discussion. The fourth session (D + 1 year) opened with participants completing the questionnaire. Next, participants in the cancer arm received information about oral hygiene, while those in the control arm were shown the slideshow about cancer screening. This last session ended with a discussion, and each participant received a diploma. At the end of each session, a procedural compliance sheet (Han et al., 2022; Sanetti et al., 2021) was completed to verify session completeness. This form also indicated the general atmosphere, the participants’ attention, and possible disruptive events (Fig. 3).

### Involvement of persons with ID in the design and conduct of the research

Persons with ID were invited to collaborate at various stages of the research, from before initiation to after closure (Supplementary methods).

### Outcomes measurement

In both groups, a 20-item questionnaire was used to evaluate knowledge of cancer (score 1) and intention to participate in cancer screening (score 2). This questionnaire enabled indirect estimation of participants’ understanding and memorization of cancer screening information at the four measurement times: D-15 days, D0, D + 3 months, and D + 1 year. A rating of 0–10 was assigned for each score.

### Statistical analyses

We performed analyses to compare the two intervention groups at different assessment times. Qualitative variables are presented as numbers and percentages, and quantitative variables as mean and standard deviation. Mixed linear regression with a random intercept on the cluster enabled the comparison of scores between groups at different times. Significance estimate correction was performed with the method of Kenward and Roger (10.1136/bmj.k1121), considering the increased alpha risk in analyses of fewer than 40 clusters. Multivariate mixed linear regression analyses were conducted to account for residual confounding factors linked to imbalances in patient profiles within these clusters. We estimated the evolution from baseline scores 1 and 2, comparatively between the two groups, and with adjustment for age, gender, and deficit level. Statistical analyses were performed using R software version 4.1.1.

## Results

The experimental group included 19 clusters (306 individuals; age range: 20–76 years). The control group included 19 clusters (302 individuals; age range: 20–75). The mean age was 42 years in both groups. The experimental and control groups were similar in terms of sex, age, ID level, evaluation of questionnaire difficulty, and need of help to answer the questionnaire (Table 1).

**Table 1:** Description of the sample

Characteristics	Experimental, *N* = 306^1^	Control,* N* = 302^1^
**Sex, male**	166 (54%)	190 (63%)
**Age**		
> 50 years	88 (29%)	98 (32%)
25–50 years	182 (59%)	172 (57%)
< 25 years	36 (12%)	32 (11%)
**Intellectual disability**		
Mild	181 (59%)	159 (53%)
Moderate	125 (41%)	143 (47%)
**Score 1, mean ± SD**	5.06 ± 2.42	5.26 ± 2.53
**Score 2, mean ± SD**	5.1 ± 3.3	4.7 ± 3.4
**How were the questions** **answered?**		
No answer	7 (2.3%)	13 (4.3%)
With help	189 (62%)	149 (49%)
Alone	110 (36%)	140 (46%)
**Questions were deemed**		
Very easy	102 (33%)	67 (22%)
Easy	127 (42%)	158 (52%)
Hard	55 (18%)	53 (18%)
Very hard	18 (5.9%)	20 (6.6%)
No answer	4 (1.3%)	4 (1.3%)

^1^*n* (%)

**Fig. 4:**
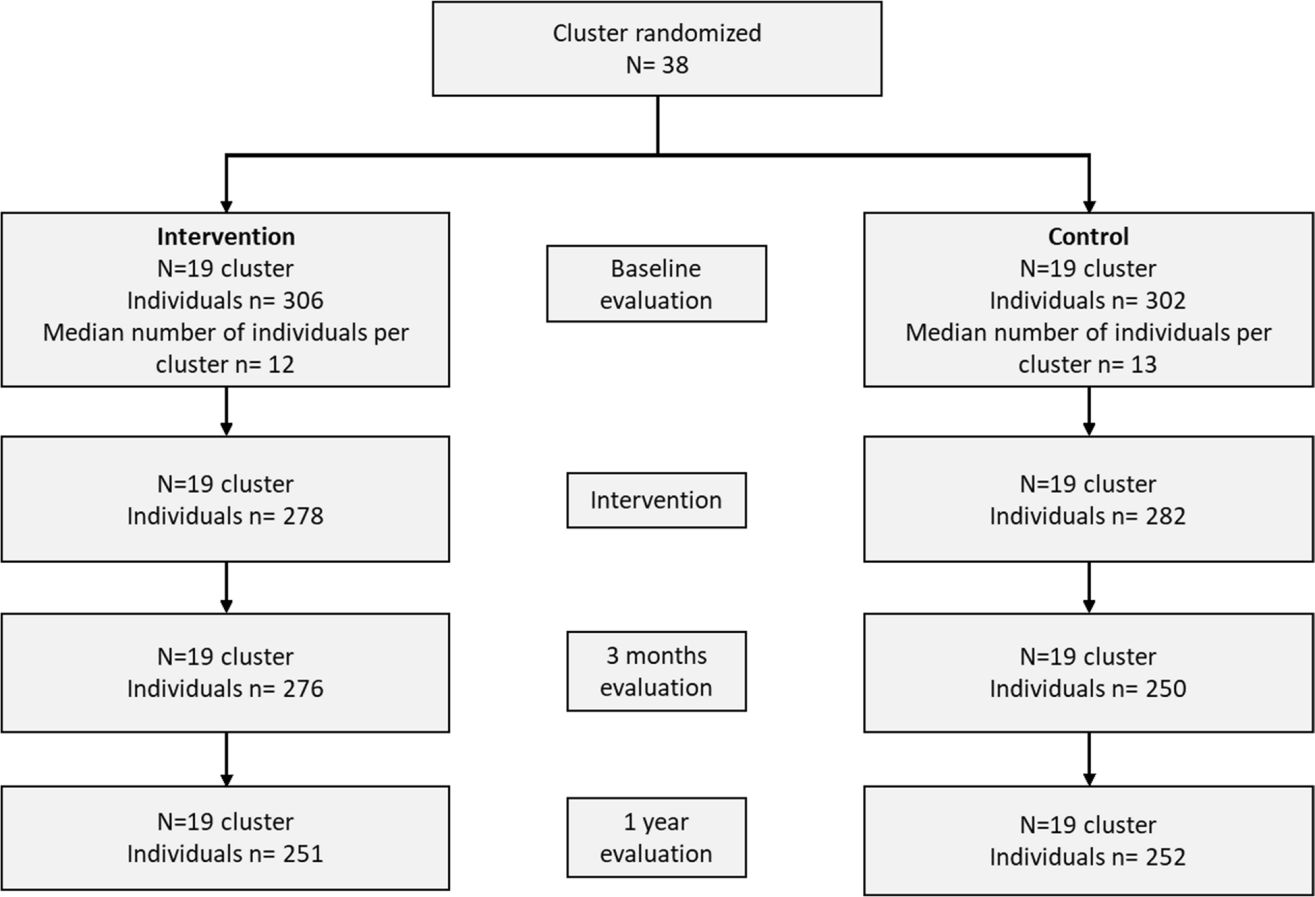
Flowchart of study participants

The study began with 608 participants. Of these participants, 85 (14%) left the study due to health problems or institutional or family constraints, and 18 (3%) left following a personal decision. With these drop-outs, the study included 560 participants at D0 (− 8%), 526 at D+3 months (− 14%), and 503 at D+1 year (− 17%) (Table 1, Fig. 4).

### Primary outcome: Understanding and memorization

For the experimental group, on the intervention day (D0), we observed significant increased knowledge of cancer and cancer screening (score 1) (*p* < 0.001), and intention to participate in cancer screening (*p* < 0.001). At 3 months, the same group showed a significant change in memorization of knowledge (*p* < 0.001), but no significant change in intention to participate in screening (Table 2).

**Table 2:** Outcomes at D0, D + 3 months, and D + 1 year

Score evolution (mean ± SD)	Experimental	Control	*p*-value
**D0**	*N* = 278	*N* = 282	
Score 1	2.26 ± 2.27	0.44 ± 1.83	< 0.001
Score 2	2.1 ± 3.6	0.3 ± 3.4	< 0.001
**D + 3 months**	*N* = 276	*N* = 250	
Score 1	1.51 ± 2.10	0.61 ± 2.09	< 0.001
Score 2	1.1 ± 3.6	0.7 ± 3.5	0.26
**D + 1 year**	*N* = 251	*N* = 252	
Score 1	1.48 ± 2.08	0.69 ± 2.04	< 0.001
Score 2	1.2 ± 3.7	0.5 ± 3.5	0.068

Complementary analyses indicated that understanding and intention to participate in screening did not significantly differ between young and old participants, participants with mild and moderate ID (Table 3).

**Table 3:** Evolution of scores from baseline, after multivariate adjustment

Score evolution from baseline		Score 1	Score 2
Sex	Men	-	-
	Women	− 0.44 (− 0.73 to − 0.14, *p* = 0.002)	− 0.09 (− 0.60 to 0.42, *p* = 0.365)
Age	> 50 years	-	-
	25–50 years	0.30 (0.01 to 0.61, *p* = 0.027)	0.06 (− 0.46 to 0.58, *p* = 0.408)
	< 25 years	0.65 (0.15 to 1.15, *p* = 0.006)	− 0.10 (− 0.95 to 0.75, *p* = 0.409)
Intellectual disability	Mild	-	-
	Moderate	0.01 (− 0.30 to 0.33, *p* = 0.470)	0.13 (− 0.39 to 0.65, *p* = 0.307)
Time	15 min after intervention	-	-
	3 months after intervention	− 0.29 (− 0.44 to − 0.13, *p* < 0.001)	− 0.27 (− 0.54 to − 0.00, *p* = 0.023)
	1 year after intervention	− 0.22 (− 0.38 to − 0.07, *p* = 0.003)	− 0.28 (− 0.56 to − 0.01, *p* = 0.021)
Group	Control	-	-
	Experimental	1.18 (0.78 to 1.58, *p* < 0.001)	1.01 (0.43 to 1.59, *p* < 0.001)

### Secondary outcomes

At one year, participants still exhibited memorization of knowledge (*p* < 0.001), and there was a trend towards greater intention to participate in cancer screening in the experimental group compared to the control group (*p* = 0.068). Subgroup analysis among men revealed a significantly worse score 2 in the control group compared to the experimental group (difference of − 1.47 points; − 2.12 to − 0.81; *p* < 0.001). In contrast, among women, we observed no significant difference between the control and experimental groups (difference of − 0.32 points; − 1.09 to 0.46; *p* = 0.214.

The results were not influenced by participants’ place of residence. The mean difference in score 1 between the tested versus control group was 1.41 ± 1.87 for participants living in institutions (*n* = 200) and 1.43 ± 2.17 for those living with their family or in the community (*n* = 87). The mean difference in score 2 between the tested versus control group was 1.2 ± 3.5 for those living in institutions, and 1.1 ± 3.8 for those living with their family or in the community.

On the procedural compliance sheet, using a 10-point scale, the atmosphere was estimated as 8.09, participant involvement as 8.25, and participant attention as 7.91 on average, for the experimental group. The corresponding ratings in the control group were 8.26, 8.22, and 8.04, respectively.

### Qualitative outcomes

Study participants showed great interest in the research, evidenced by the low attrition rate, with only 18 voluntary abandonments over one year. It is not possible to determine whether the fidelity in the experimental and control groups was due to the provision of final information on oral hygiene and cancer, respectively. It is also not possible to ascertain whether the diploma awarded to each participant contributed to their loyalty. This reward recognized their value and was very appreciated (Fig. 3).

### Involvement of participants with ID

From the beginning, pilot group members with ID gave advice regarding the most appropriate way to conduct the study. They suggested that information should be provided by health providers rather than professional caregivers, explaining that physicians and nurses inspire more confidence as validated professionals. They also recommended a maximum session length of 1.5 h and suggested including a break time to help maintain good attention throughout the session. Additionally, they modified the PowerPoint presentation, indicating how messages should be explained in the slideshows about cancer prevention and oral hygiene.

Pilot group members with ID carefully read the questionnaire and corrected questions that were either too simple or too difficult to understand, and suggested the deletion of text that too strongly suggested the good answer. Before the study began, people with ID contributed to the booklet about cancer screening in persons with ID. Pilot group participants with ID, and actors in the film, provided suggestions for clarifying the message and making it more incentivizing. For example, they suggested appealing to the viewer by saying “you too should get tested” Moreover, pilot group members with ID provided ideas about how to recruit their peers, and designed a poster that was sent to each institution involved in this study. At the end of the study, persons with ID gave advice regarding how to broadcast the study results, and highlighted the need to present these results to professional caregivers. One person with ID accompanied the team to a presentation of the study results at a conference. Importantly, they also helped ensure the inclusion of a sufficient number of participants in the study. Their contributions fulfilled our expectations and improved the study tools, films, workshops, and questionnaire.

## Discussion

Few studies have evaluated the effectiveness of providing health education and information about cancer—and particularly cancer screening—in persons with ID (Gil et al., 2024; Parish et al., 2012; Swaine et al., 2014; Walsh et al., 2021; Wilson et al., 2018). The present study extends previous work. We demonstrated that an intervention combining different educational approaches, which mobilize visual, auditory, palpation, games, and discussion skills, including in persons with low or no literacy (Petitpierre et al., 2025), successfully improved participants’ understanding of cancer screening. This is the first study to measure progress in knowledge up to one year after training.

The low attrition rate after one year supports the feasibility of the utilized protocol, and indicates that people with ID are interested and desire involvement in their own health. Participants did not receive financial compensation for their contributions, and thus were likely motivated to participate due to interest in the study. The successful completion of this study was largely due to the inclusion of persons with ID, starting from the study design, and continuing throughout its completion. Their involvement helped us to avoid making mistakes due to ignorance of their intellectual potential, their expectations in terms of health, and their ways of understanding and thinking about health topics. Their contributions enabled the successful realization of this study.

A review of 22 studies evaluated effective health education for people with ID, and found that learning was reinforced by 1) embedded program flexibility, 2) appropriate and accessible resources, 3) motivating delivery, as well as 4) effective context, 5) a supportive environment, 6) and long-term opportunities (Owens et al., 2020). On the basis of our study, other facilitating factors include small groups of ten persons or less, and the arrangement of discussion time between trainers and participants, as well as between participants. Along with Geukes et al. (2019) we have found that health learning is not individualistic, but rather strongly dependent on social interactions.

The participants gained important knowledge during discussions after the slideshow, during workshops, and after viewing the film. At these times, rather than passively listening to the message, participants became actively engaged in discussions with the presenters and each other. This confirms Vygotski’s theory (Vygotski, 1978) that concept formation stems from discussions (Guerrin et al., 2012) between persons. Workshops during which the participants saw and palpated plastic organs also importantly contributed to their understanding, as predicted by the Piagetian theory (Bourgeois, 2018).

A complementary evaluation is planned for five years after the study’s end, to measure screening attendance in the institutions, and to analyze breast and colon cancer stage at diagnosis, through use of the Hérault cancer registry.

### Limitations and strengths

This study has several limitations. First, it included persons who were either living or working in institutions for persons with ID. It did not evaluate persons with ID who were living in the community or with their family. Similarly, it did not include persons with severe ID. Thus, our results cannot be generalized to all persons with ID. Second, the participants were not randomly chosen, but were rather recommended by caregivers at each institution, who selected persons able to follow the intervention and those who volunteered to participate. Third, the observed gain of understanding does not imply that the participants know all about cancer screening, but only that they understood and memorized what the research team estimated were the most important things about cancer screening. Finally, although much care was taken to avoid fears and anxiety, we did not measure the anxiety and fears about cancer that may have been raised by the sessions.

A main strength of this study was its development as a randomized controlled trial including the largest sample of adults with ID among studies of this topic. The sample size was calculated to elucidate differences between participants of the experimental versus control group. Furthermore, understanding and memorization were evaluated up to one year post-intervention. Moreover, the two groups’ sessions were similar, except for the health content. Additionally, there was no contact between the experimental and control group due to the cluster structure of the trial. Notably, the conduct of sessions and the questionnaire development were guided by an academic researcher with experience teaching people with special needs. Another important strength is that this study included and greatly benefited from help from persons with ID.

## Conclusion

This study contributes to evidence that people with mild and moderate ID can learn and retain health information on cancer. The findings also suggest that people with ID have greater understanding and memorization capacity than is usually imagined. The low attrition rate indicates that the present method is feasible and reproducible as conducted. It also illustrates that people with ID are interested in and desire active involvement in their health. The participation of people with intellectual disabilities in the steering group was crucial for the successful completion of this research.

## “So, What” Contributions to knowledge

What are the innovations in this policy or program?

- This study applied a new model, mobilizing different learning methods—including visual, auditory, tactile manipulation, and allowing ample time for discussion.
- Persons with intellectual disability helped with this study by checking and modifying various tools (questionnaire, PowerPoint presentations, films, and games), and giving advice about recruiting participants and conducting sessions.
- The positive result of this study, which included 608 participants, supports the efficacy of actions conducted directly among people having intellectual disability, and with the help of people with intellectual disability, to improve cancer screening in this population.
- The study was underpinned by three theories of learning: Piaget’s (direct experimentation and manipulation) Bandura’s (modeling and identification of opportunities), and Vygotski’s (formation of knowledge through human relationships).

What are the key implications for public health interventions, practice, or policy?

- The diversification of learning methods—particularly to include greater time for moments of discussion—seems to increase learning performance***.***
- Stronger involvement of people with intellectual disability in the construction of this program, and in the progress of each stage, enabled use to increase its effectiveness***.***
- Before expanding this method, it is necessary to measure whether this training has an impact on anxiety among the people with intellectual disability.

## Supplementary Information

Below is the link to the electronic supplementary material.

ESM 1 (DOCX 20 KB)

ESM 2 (PDF 3.57 MB)

ESM 3 (DOCX 24 KB)

## Data Availability

Data are available. Contact Dr Daniel Satgé.
