# Supplementary material for: Cancer screening learning for adults with intellectual disability
Source: Can J Public Health. 2026 Apr 29;117(Suppl 1):94–103. doi: 10.17269/s41997-025-01105-6 (PMC13129136; doi:10.17269/s41997-025-01105-6)
Supplement: Supplementary file 1 — (DOCX 20 KB) [file 41997_2025_1105_MOESM1_ESM.docx]

**Supplement**

**Cancer screening education for adults with intellectual disability: A randomized controlled trial**

**Supplementary methods**

**Size of the target sample**

The initial hypothesis was that the pre-intervention score and 3-month post-intervention score would differ by at least 2 points between the intervention and control groups, with a maximum pessimistic standard deviation of 2.5. Thus, 25 participants per group had to be included, for a power of 80% and a two-sided alpha risk of 5%. To account for the data correlation induced by cluster randomization, the sample size was increased by applying an inflation factor defined by (1 + [m − 1] *ρ), where m is the average establishment size, and ρ is the correlation level between the responses of people in the same establishment. Assuming a conservative correlation coefficient ρ of 0.5, and an average facility size of 20 participants, we obtained an inflation coefficient of 10.5, suggesting an increase of 263 participants per group. After applying a conservative drop-out rate of 30%, the final estimate of the number of participants needed was 342 per group (684 total participants).

**Randomization procedure and group characteristics**

For organizational reasons, cluster randomization was preferred over individual randomization. Cluster randomization (1:1 ratio), was performed before the intervention (D0), using a computer-generated list of random numbers. Each participant was assigned a number indicating their study arm, a cluster number, and an individual number within the cluster, using R software and the “blockrange” package. Ultimately, the experimental group and control group each comprised 19 clusters (306 and 302 individuals, respectively).

The control group was carefully designed to be similar to the tested group, except for the content of the message. The groups had the same trainers, same small number of participants, same session length, and sessions held in the same type of room. The PowerPoint presentations were of the same length, the films were nearly the same length, and the participants in the control group also received a booklet on oral hygiene. Additionally, the same time was given for discussion in both groups.

**Advisory group**

The advisory group included 11 persons who were chosen for their knowledge regarding intellectual disability (ID) and their interest in the health of persons with ID. The group included an academic researcher whose work focuses on people with special needs (group leader), two persons with ID from the “Nous Aussi” (We Also association), a physician, the director of a medico-social institution, a caregiver, two nurses, the two researchers who conducted the study, and the principal investigator. These persons received no payment.

**The main common didactic principles of the interventions**

Briefly, the learning interventions were constructed based on three main learning theories: the Piagetian theory, which considers that learning and knowledge are acquired through direct experience and concrete manipulatives, rather than passive receipt of information from a teacher (Bourgeois, 2018); Bandura’s theory, which underlines that modeling and identification opportunities may favor openness to new behaviours and/or attitudes (Guerin, 2012); and Vygotski’s socio-cognitive approach, which situates the knowledge formation within the relationship between human beings, particularly while exchanging ideas and thoughts (Vygotski, 1978). These foundations led us to conduct our training modules in small groups of persons with ID (rather than in a one-to-one setting), to include workshops and a game, to prepare and show a film allowing identification, and to devote significant time to discussions.

**Questionnaire development**

Since no questionnaire was available to evaluate knowledge of cancer and cancer screening, the utilized questionnaire was created especially for the study. It included the same pictures from the booklet on cancer screening, “*Monique is having a mammogram, Gilles does a colorectal test, Leila does a smear*”, which was created by Oncodéfi. The twenty questions evaluated civility and sex, knowledge (i.e. score 1 in the study), and intention to participate in cancer screening (score 2). The questionnaire was evaluated and modified based on a document created by the research team (particularly by an academic teacher for people with special needs), and was then validated by participants with ID.

**Detailed involvement of persons with ID in the design and conduct of the research**

When developing the program, pilot group participants with ID helped assess the feasibility of the sessions, with regards to total study length, and optimal session duration. Persons with ID also actively participated in the creation of communication supports. They co-constructed the screening booklet that was available before the beginning of the study; were asked to revise the film scenario and to act in the film that explains organized cancer screening; and were invited to evaluate the slideshow and questionnaire. Additionally, the texts of the booklet, the slideshow, and the questionnaire were revised by a team expert in “Easy To Read And Understand” adaptation. During steering meetings, persons with ID were asked about the best way to recruit participants. Finally, persons with ID also took note of the study results, and were asked to communicate their understanding of the findings, to make proposals regarding dissemination of the study to their peers and professional carers, and to participate in academic presentations of the results.

**Procedural fidelity**

Procedural fidelity, also called treatment fidelity, describes the extent to which an intervention is implemented as planned. Procedural fidelity is a key component affecting the internal validity of research (Han et al., 2022). If an interventional study fails to report the treatment fidelity, or reports low treatment fidelity, the study findings may be difficult to interpret, because the intervention may not have been implemented as planned (Sanetti et al., 2021). We assessed our procedural fidelity using a dichotomous scale (yes/no), for each of the nine steps planned in the cancer screening teaching module: introduction and greetings, PowerPoint presentation, workshop 1 (breast cancer screening), workshop 2 (cancer games), workshop 3 (blue envelope with colorectal kit), 10-min break, questionnaire, booklet distribution, and conclusion of the session.

The control groups (*n*=29) included an average of 9.41 participants (range: 5–17). Of the 29 groups, 26 (89.65%) received the complete dental hygiene training (all 8 steps), two groups (6.89%) did not receive one step of the training, and one group (3.44%) did not receive two steps. The main reason for groups missing steps was lack of time (room had to be freed up, workshop took a little longer than planned, etc.). The average number of steps completed was 7.86 out of 8.

The experimental group (*n*=34) included an average of 8.32 participants (range: 5–13). Of the 34 groups, 31 (91.17%) received the full cancer screening training (all 9 steps), three groups (8.82%) did not receive one step of the training. There were three distinct reasons for missing steps: lack of time (closure of the establishment), refusal by one group to take the break, and the “breast” workshop was not presented in a group comprising only men. The average number of steps completed was 8.91 out of 9.
